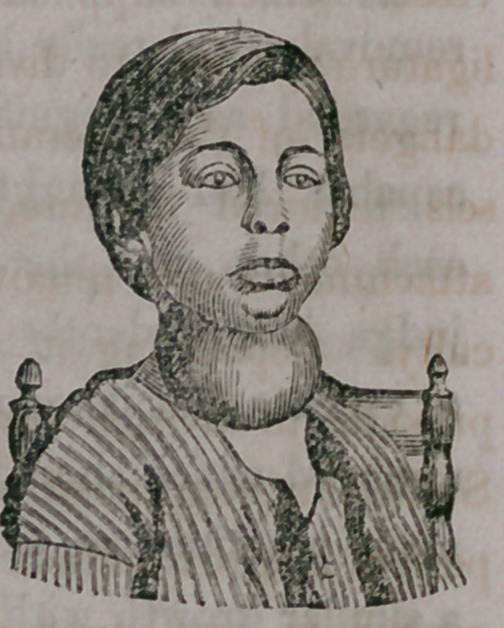# Surgical Cases—Sero-Cystic Degeneration of the Mammary Gland and Cystic Tumor in Neck, with Wood Cut Illustrations

**Published:** 1864-05

**Authors:** J. F. Miner


ART. III.—*Surgical Cases—Sero-Cystic Degeneration of the Mammary Gland and Cystic Tumor in Neck, with wood cut Illustrations.* By J. F. Miner, M. D.

Cystic Tumor of Breast weighing 15 lbs.

Mrs. Wm. E. Isham, of Decatur, Michigan, aged 34, of natural delicate constitution, first noticed about ten years since that her left breast was enlarging; was hard, and tender. The progress of the disease was at first very slow, and but little attention was given to it; at length it had become so much increased in volume as to annoy her, more from the bulk and apprehension, than from actual pain. It continued gradually but constantly to increase, though a great many applications were made and many surgeons consulted, both in the west and in the eastern cities. Cancer Doctors and Purifiers of the Blood had also secured the usual attentions paid by subjects of such disease, but the progress was uninterrupted, and the lady had finally nearly abandoned all hope, returning to the home of her mother, in Wales, Erie County, N. Y., to die, rather than with any expectation of recovery. It had grown to the enormous dimensions represented in the illustration, extending high up into the axilla, crowding the arm back from its natural depending position, and extending downwards so as scarcely to be encircled by the two arms, which was necessary for support when locomotion was attempted. The weight of the tumor had become so great that the patient was mostly confined to the recumbent position, and where it had rested upon the bed, the pressure had produced an open ulcer, resembling in some respects a small encephaloid tumor, which had deceived many, and caused them to regard it as of a malignant character. The general health had become much impaired, more from the load, which the patient had so long carried and from the dread of impending evil, than from any actual disease or suffering; pains, however, were complained of as extending through the side, but not with sufficient severity to prevent sleep.

In this condition Dr. Cornwell, of Yorkshire, was invited to take charge of the case, and direct what efforts, if any, should still be made to obtain relief from the disease, or for allaying the sufferings of its progress and termination. Through favor of Dr. Cornwell I was invited to visit the patient, with view of operation, as he regarded it as benign in character, and possibly susceptible of removal.

March 3d, 1864.—In consultation with and assisted by Drs. Cornwell Colegrove, Wainwright and Havens, it was agreed to attempt the removal, though differences of opinion were entertained as to its practicabilty and possibility. The tumor was manifestly very vascular; enlarged veins passed over the mass in all directions. The patient could not bear the loss of much blood, but chose to risk all in the attempt to save life, rather than longer carry this "body of death." Sulphuric ether having been administered to full anæthesia, incision was made over the upper portion near the axilla where it was presumed the larger supply arteries would be found. By carefully separating this portion from its attachments and raising it, the large vessels which supplied the growth were exposed and enclosed in a strong ligature previous to division. After accomplishing this, the difficulties and dangers of the operation were passed. A large number of small vessels required ligature, still the tumor was rapidly separated from its attachments and removed, without injurious hæmorrhage, or serious difficulty. Separating it from the axillary region and securing the vessels previous to division are the only points worthy of mention in the operation. Sufficient integument was dissected from the sides of the tumor to allow of perfect approximation. Thus easily was removed a growth which appeared to rest upon so large a base, possess so great vascularity and extend into the important surgical region of the axilla so deeply, as to be beyond safe removal. Cicatrization took place rapidly, and the patient recovered without unpleasant symptom, as reported by attending physicians Drs. Cornwell and Wainwright.

The mass, upon examination after removal, was found to be made up of a great many cysts, with considerable cellulo-fibrous material interposed between; the different cells or cysts, containing dissimilar fluid, mostly of a serous nature, but of various colors, constituting what was formerly called "*Cystic Sarcoma,*" a disease of benign character, and in no way liable to reappear after removal.

Tumors of this character are rarely met with in the breast, and are still more uncommon of the size which this had attained. Hypertrophy of the breast is said to exceed in some rare instances this immense growth, which weighed after the escape of the contents of several cysts and of the vessels, fifteen pounds. The gland itself was not much involved in the disease, which appeared to have commenced from its lower border, and continued an independent growth.

The breast, ovary and testicle appear to be exposed to nearly the same forms of disease; cystic degeneration of these organs is at least met with, which is identical in general character, and only modified by the different structures or circumstances of growth. Specimens of this disease from all these organs have been obtained and preserved within the last few months, and upon comparison every observer would at once regard them as of the same family—a very troublesome family oftentimes.

Similar disease may locate upon other organs, or may appear as an independent growth, but cystic degeneration is the most common disease of the ovary, frequent in the testicle, and sometimes affects the breast; whenever located upon any of these organs it possesses the same general characters.

*Cystic Tumor of the Neck.*—The annexed cut represents the appearance of a boy 10 years old, son of Charles Cockle, of Evans, who was presented for operation April 2d, 1864. This tumor commenced to grow, or was distinctly perceivable when the boy was six months old, and had gradually increased in size until the pressure was so great upon the trachea as to cause difficulty of respiration and a peculiar whistling croupy sound often heard in the adjoining room when the patient was sleeping with the face turned upwards. It was found on removal to weigh eight ounces, and to be composed of a fibrous cyst containing a thick, yellow, oily fluid, with a great abundance of the glistening crystals of cholesterin, hence of the character named by Cruveilheir "laminated, nacreous, fatty tumor." The cyst was most intimately connected with the surrounding tissues, which appeared to consist wholly of vessels admitting of no dissection without hæmorrhage. With care the tumor was enucleated and removed without serious difficulty, and the patient returned home in eight or ten days nearly well.

The points of interest in the case are its location, character, period of growth, and size, rather than anything in the rarity of such disease, or the difficulty of removal. "Oh," says one, "It is nothing but an encysted tumor; such things are of common occurrence and are very easily removed."

This is just what I propose to say; not quite so common, however, and not always so readily removed. It is but a few months since we were invited to witness an operation for the removal of a similar growth, of perhaps half the size of the one here represented which was similarly situated upon the neck of a lady in middle life. Dr. March, of Albany, had been invited to make the operation; while on the part of friends there was great show of preparation. The tumor was cut down upon and removed. It had scarcely any vascular connections; was clearly an extra growth. and had little appearance of being connected with the thyroid gland, or of being an enlargement of that organ. We observed with pleasure the removal of the growth, but as no remarks were made explanatory or otherwise, returned with the private conviction that we had been "sold"—that an operation of little importance had been magnified five hundred diameters.

Upon inquiry, however, the mystery was readily solved. The growth had been regarded as a glandular enlargement, and the most distinguished surgeons of New York City had been misled, and refused to attempt its removal—had really been the magnifying power, and consequently it was regarded as a magnificent thing, to accomplish what so many of the most capable had thought difficult or impossible. Surgeons had blinded each other, and there was great honor reserved for the one who could judge independently and act accordingly. There was no skill requisite to execute, but it required courage to plan. It seemed as nothing when removed which should have importance attached to it, and I have no doubt many practitioners, without consultation and without hesitation, would remove a similar growth with the assistance simply of a neighboring boy.

The history, in outline, which is given of one case, will answer, in many respects for both; at least will show that the case is not without interest, in addition to its being remarkable in size, character, growth, and the effects it produced upon respiration.

## Figures and Tables

**Figure f1:**
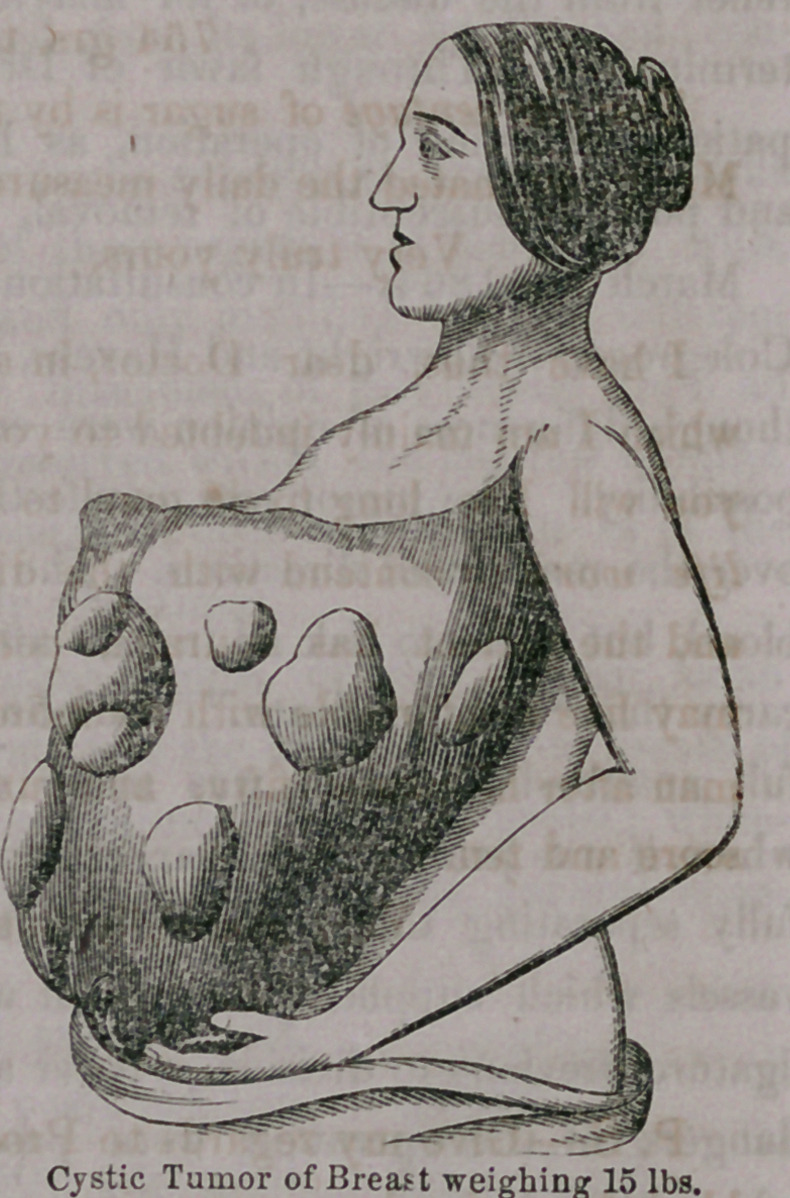


**Figure f2:**